# The Interplay Between IL‐6, Hepcidin, and BMPR2 Signalling in Pulmonary Arterial Hypertension: Mechanistic Insights Into Vascular Remodelling

**DOI:** 10.1002/pul2.70272

**Published:** 2026-04-05

**Authors:** Quezia K. Toe, Theo Issitt, Gregory J. Quinlan, S. John Wort

**Affiliations:** ^1^ Faculty of Medicine, Imperial College London National Heart and Lung Institute London UK; ^2^ Adult Centre for Pulmonary Hypertension Royal Brompton Hospital London UK

## Abstract

Pulmonary arterial hypertension (PAH) is characterized by excessive pulmonary vasoconstriction and vascular remodelling, with mutations in bone morphogenetic protein receptor type 2 (BMPR2) being the most common genetic alteration associated with the disease. While inflammatory mediators like interleukin‐6 (IL‐6) and the iron‐regulatory hormone hepcidin have been implicated in vascular remodelling, their interaction with BMPR2 signalling remains poorly understood. This study investigated how IL‐6 and hepcidin influence BMPR2 expression and downstream signalling in human pulmonary arterial endothelial cells (hPAECs). Using qPCR and Western blot analyses, we demonstrated that both IL‐6 and hepcidin significantly reduced BMPR2 mRNA and protein levels in hPAECs. Intriguingly, despite this reduction, SMAD1/5 phosphorylation remained active, suggesting compensatory signalling through alternative receptor complexes. Treatment with IL‐6 and hepcidin upregulated inhibitors of differentiation (ID) protein expression, mimicking the effects observed with BMPR2 knockdown. These findings reveal a novel regulatory axis involving IL‐6, hepcidin, and BMPR2 in PAH pathogenesis, where IL‐6 and hepcidin promote vascular remodelling through both BMPR2‐dependent and independent mechanisms. These results suggest that therapeutic strategies targeting this axis, particularly those aimed at rebalancing BMP/TGF‐β signalling, may hold promise for treating PAH.

## 1 Introduction

Pulmonary arterial hypertension (PAH) is a progressive and multifactorial disease characterized by excessive pulmonary vasoconstriction and pathological vascular remodelling. This remodelling involves all layers of the pulmonary vasculature (intima, media, and adventitia), ultimately reducing the cross‐sectional area of pulmonary arteries, increasing right ventricular afterload, and impairing cardiac performance [1, 2, 3]. Ultimately, this remodelling leads to right heart failure, a primary cause of morbidity and mortality in PAH.

Central to the pathogenesis of PAH is the dysregulation of the bone morphogenetic protein receptor type 2 (BMPR2), a member of the transforming growth factor‐beta (TGF‐β) superfamily [4, 5]. Mutations in BMPR2 are the most common genetic defect associated with familial PAH, accounting for 53%–86% of familial cases and 14%–35% of idiopathic cases [6]. Beyond mutations, reduced BMPR2 signalling is also observed in idiopathic PAH patients without detectable BMPR2 variants, highlighting the complexity of BMPR2‐related mechanisms in PAH [4]. However, BMPR2 mutations demonstrate incomplete penetrance, implicating additional genetic or environmental “second hits” in disease manifestation [5].

Recent research suggests that an imbalance between BMPR2 and TGF‐β signalling contributes significantly to PAH pathogenesis. BMPR2 acts as a molecular gatekeeper, balancing TGF‐β and BMP signalling to regulate key processes like vascular homeostasis, proliferation, and extracellular matrix remodelling [7]. This balance is disrupted in PAH, with BMPR2 deficiency leading to alternative signalling through mixed BMP/TGF‐β receptor complexes and promoting pathological responses [7]. Moreover, endothelial cells expressing mutant BMPR2 exhibit heightened susceptibility to apoptosis and release factors that enhance smooth muscle cell proliferation, further driving vascular remodelling [5, 8].

While the role of BMPR2 mutations in PAH is well‐recognized, the intersection of BMPR2 signalling with inflammatory mediators such as interleukin‐6 (IL‐6) and hepcidin remains underexplored. Our prior work has shown that IL‐6 and hepcidin influence iron homeostasis, driving pathological remodelling by promoting smooth muscle and endothelial cell proliferation and migration [9, 10, 11]. Elevated levels of IL‐6, a key pro‐inflammatory cytokine, are associated with worse outcomes in PAH, potentially through activation of pathways involving BMPR2 and hepcidin [12]. Similarly, hepcidin, the master regulator of iron metabolism, modulates vascular responses and has been implicated in pulmonary vascular remodelling [13]. Furthermore, iron homeostasis is key to normal endothelial function [14]. It was therefore our aim in this study to investigate the pathways driving the interplay between BMPR2, IL‐6 and hepcidin.

Given our previous findings, we hypothesize that IL‐6 and hepcidin contribute to PAH pathogenesis by downregulating BMPR2 and altering downstream signalling pathways. Specifically, we propose that these factors mediate vascular remodelling through changes in BMPR2‐dependent and independent signalling, with implications for therapeutic targeting. The specific aims of this study are to: 1. Assess the effects of IL‐6 and hepcidin on BMPR2 expression in human pulmonary endothelial cells (hPAECs) and 2. Evaluate downstream signalling alterations following BMPR2 modulation by IL‐6 and hepcidin.

## 2 Material and Methods

### 2.1 Cell Culture

Human pulmonary artery endothelial cells were purchased fully characterised from Promocell (Germany) and ATCC (USA); cells were purchase from two suppliers to increase the number of donors. hPAECs were maintained on endothelial cell growth medium 2 (EGM2; Promocell) supplemented with foetal calf serum (2%, FCS), epidermal growth factor (5 ng/mL), basic fibroblastic growth factor (10 ng/mL), insulin‐like growth factor (20 ng/mL), vascular endothelial growth factor 165 (0.5 ng/mL), ascorbic acid (1 µg/mL), heparin (22.5 µg/mL) and hydrocortisone (0.2 µg/mL). hPAECs cells were passaged when 90% confluent was reached. Further, the cells were cultured with fresh medium in T25/T75 flasks at 10,000 cells per cm^2^, incubated at 37°C and 5% CO2. Only cells from passage 4–8 were used in the experiments.

For hPAECs treatments heparin and hydrocortisone were omitted from the media. Heparin was excluded because it is a strong hepcidin inhibitor [15] and hydrocortisone was removed due to its anti‐inflammatory properties.

Hepcidin peptide was purchased from Peptides International (now known as vivitide, Massachusetts, USA). The lyophilised peptide was re‐suspended with double‐distilled, sterile water to the stock concentration of 1 mg/mL. Stock aliquots were made and store at −80°C for long‐term storage. Recombinant human interlukin‐6 (IL‐6) was purchased from GIBCO, USA, the carrier‐free lyophilised protein was reconstituted with 100 mM acetic acid to a concentration of 100 μg/mL. The reconstituted protein was apportioned into working aliquots and stored at −20°C.

### 2.2 siRNA Transfection

BMPR2 silencing in hPAECs was induced using siRNA. Briefly, hPAECs were transfected with 50 nM ON‐TARGETplus SMARTpool siRNA (Horizon Discovery, UK) against BMPR2 (siBMPR2), or a non‐targeting control pool (siControl), complexed with Lipofectamine RNAiMAX Transfection Reagent (Thermo Fisher Scientific, UK), in Opti‐MEM Reduced Serum Medium (Gibco, UK) for 14 h. Media was replaced with EGM2 following incubation with transfection mixes.

### 2.3 Real Time PCR

Total RNA was extracted from cells using the RNAeasy Mini preparation Kit (Qiagen, UK). To determine the concentration and purity of RNA the nano‐drop spectrophotometer was used. RT‐PCR was performed using SensiFast SYBR Green Lo‐ROX (Meridian Biosciences) with primers below.

**Table:** 

Gene	Forward	Reverse
SMAD5	GATCTCACTATGTTGCTCAG	GTCCTGTAATCCCAGTTATTC
SMAD1	TCAACAGAGGAGATGTTCAG	AATTCCGGTTAACATTGGAG
BMPR2	AACAACAGCAATCCATGTTC	TATCTGTATACTGCTGCCATC
β‐actin	GACGACATGGAGAAAATCTG	ATGATCTGGGTCATCTTCTC
ID1	CCGGTCTCATTTCTTCTCGT	TCGGTCTTGTTCTCCCTCAG
ID2	ATGAAAGCCTTCAGTCCCGT	TTCCATCTTGCTCACCTTCTT
ID3	TCATCTCCAACGACAAAAGG	ACCAGGTTTAGTCTCCAGGAA
ID4	TGAACAAGCAGGGCGACA	CGTGCAAAGAAAGAATGAAAG
HAMP	GTTTTCCCACAACAGACG	CTTTGATCGATGACAGCAG
GAPDH	ACAGTTGCCATGTAGACC	TTGAGCAGGGTACTTTA

### 2.4 Western Blot

hPAECs were lysed with radioimmunoprecipitation assay buffer (supplemented with protease and phosphatase inhibitors) after 24 h treatments with IL‐6 and hepcidin. Bradford assay was performed to quantify protein and 50 μg of protein was separated on 4%–15% sodium dodecyl sulfate‐polyacrylamide gel electrophoresis and transferred onto nitrocellulose membranes. Western blot analysis was performed using mouse anti‐BMPR2 (1:500; BD Biosciences), rabbit anti‐p‐SMAD1/5 (1:500; Cell Signalling), rabbit anti‐ID1(1:500; Cell Signalling) and α‐tubulin (1:2000; Cell Signalling). Membranes were imaged with the Odyssey FC imaging system.

## 3 Results

### 3.1 IL‐6 and Hepcidin Reduce BMPR2 Expression in hPAECs

**Figure 1:**
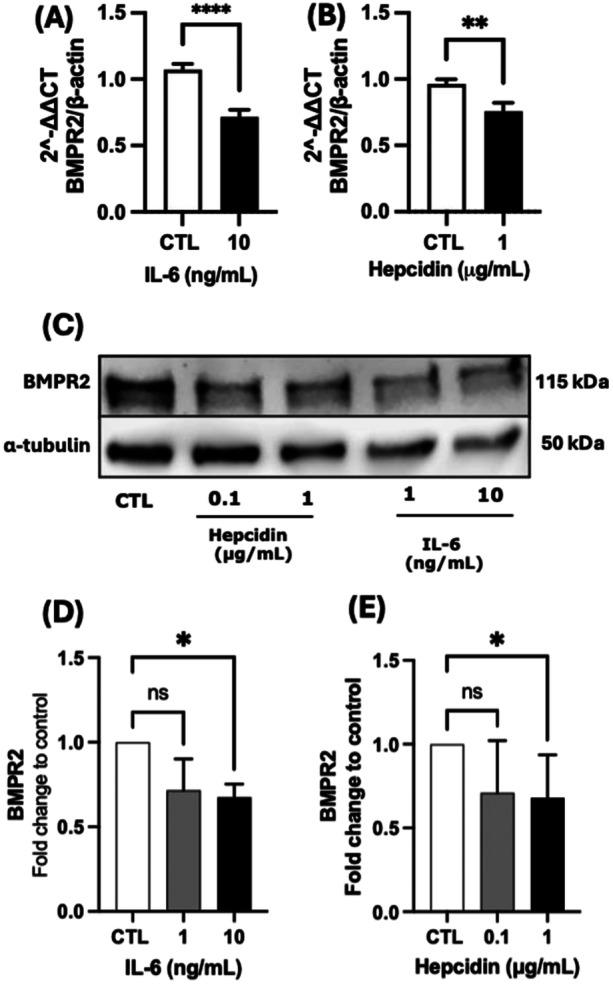
BMPR2 expression is reduced by IL‐6 and hepcidin. mRNA transcription after 24 h treatment with IL‐6 (A) and hepcidin (B), *p < 0.05. Data shown are mean ± SEM *n* = 6. Unpaired *t*‐test was performed **p < 0.01;****p < 0.0001. (C) Western blot image of BMPR2 expression on hPAECs treated with hepcidin and IL‐6. BMPR2 western blot bands relative density of hPAECs treated with hepcidin (D) and IL‐6 (E) compared to untreated control. Data shown are mean ± SEM *n* = 4. Kruskal–Wallis test followed by Dunn's post hoc test was performed *p < 0.01.

To assess the impact of IL‐6 and hepcidin on BMPR2 expression, hPAECs were treated with IL‐6 (10 ng/mL) or hepcidin (1 µg/mL). Quantitative PCR analysis revealed that IL‐6 significantly decreased BMPR2 mRNA levels to approximately 70% of control values (*p* < 0.0001) (Figure 1A). Similarly, hepcidin treatment resulted in a significant reduction in BMPR2 mRNA transcription, decreasing levels to approximately 75% of control (*p* < 0.01) (Figure 1B).

To confirm these findings at the protein level, Western blot analysis was performed on hPAECs treated with increasing concentrations of IL‐6 (1 and 10 ng/mL) or hepcidin (0.1 and 1 µg/mL) for 24 h (Figure 1C). Using α‐tubulin as a loading control, densitometric analysis revealed significant reductions in BMPR2 protein expression. Hepcidin treatment showed a concentration‐dependent effect, with 1 µg/mL reducing BMPR2 protein levels by approximately 40% compared to control (*p* < 0.01), while the lower concentration of 0.1 µg/mL showed no significant effect (Figure 1D). IL‐6 treatment demonstrated similar potency, with both 1 and 10 ng/mL concentrations significantly reducing BMPR2 protein levels by approximately 35% (*p* < 0.01) (Figure 1E). Statistical significance was determined using the Kruskal–Wallis test followed by Dunn's post hoc test for multiple comparisons.

### 3.2 BMPR2 Signalling Shows Dynamic Response to IL‐6 and Hepcidin Treatment

**Figure 2:**
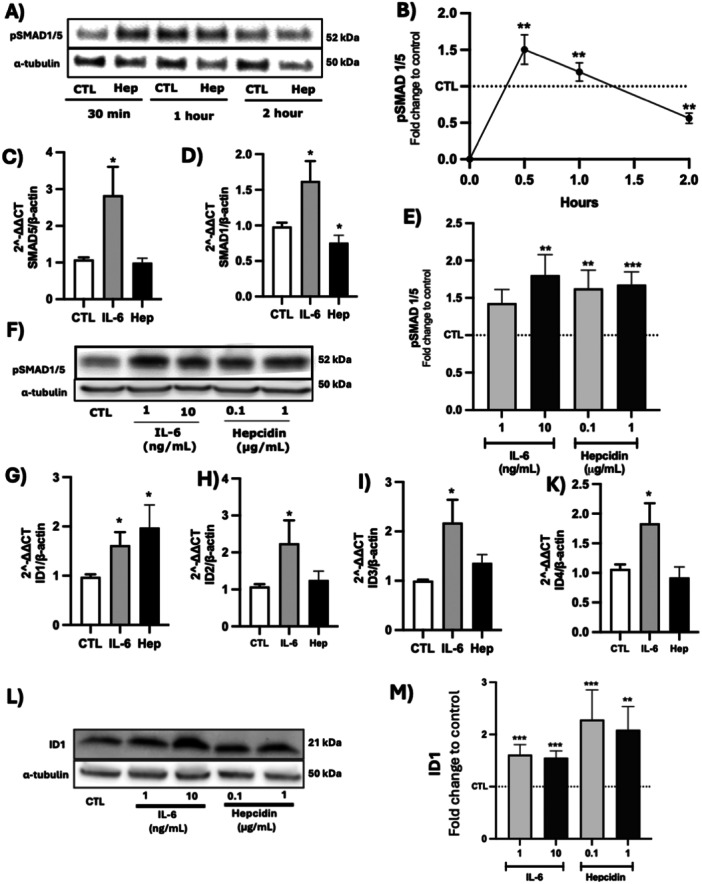
IL‐6 and hepcidin effect on BMPR2. (A and B) Western blot image and densitometry analysis of pSMAD1/5 phosphorylation expression time course after treatment with 1 µg/mL hepcidin. Data shown are mean ± SEM *n* = 5. Kruskal–Wallis test followed by Dunn's post hoc test was performed **p < 0.01. (C and D). SMAD5 and SMAD1 (D) mRNA transcription after 3 h treatment with IL‐6 (A) and hepcidin (B), Unpaired *t*‐test was performed *p < 0.05. Data shown are mean ± SEM *n* = 6. (E and F) Densitometry analysis and western blot image of pSMAD1/5 phosphorylation expression 24 h after treatment with IL‐6 (1 and 10 ng/mL) and hepcidin (0.1 and 1 µg/mL). Data shown are mean ± SEM *n* = 5. Kruskal–Wallis test followed by Dunn's post hoc test was performed **p < 0.01. (G, H, I, K) ID1 (G), ID2 (H), ID3 (I), and ID4 (K) after IL‐6 (10 ng/mL) and hepcidin (1 µg/mL) treatment for 3 h. Data shown are mean ± SEM *n* = 6. Unpaired *t*‐test was performed *p < 0.05. (L and M) Western blot image (L) demonstrating that both IL‐6 and hepcidin increase ID1protein expression. (M) Densitometry analysis of the western blot bands showed significant ID1 protein increase after 24 h treatment with IL‐6 (1 and 10 ng/mL) and hepcidin (0.1 and 1 µg/mL) compared to untreated controls. Data shown are mean ± SEM *n* = 6. Kruskal–Wallis test followed by Dunn's post hoc test was performed ***p < 0.001.

To determine whether BMPR2 reduction affected downstream signalling, we assessed phosphorylation of receptor‐activated SMAD proteins (SMAD1/5). Western blot analysis revealed a dynamic temporal response to hepcidin treatment, with SMAD1/5 phosphorylation showing significant increases at 30 min (1.5‐fold increase, *p* < 0.01), maintaining elevation at 1 h (1.2‐fold increase, *p* < 0.01), before declining below baseline levels by 2 h (0.5‐fold of control, *p* < 0.01) (Figure 2A,B).

Analysis of transcriptional responses showed that IL‐6 treatment significantly increased SMAD5 mRNA levels (2.8‐fold increase, *p* < 0.05) while reducing SMAD1 expression (Figure 2C,D). In contrast, hepcidin treatment decreased SMAD1 expression (*p* < 0.05) without significantly affecting SMAD5 levels.

Despite the initial dynamic response, both IL‐6 and hepcidin treatment for 24 h resulted in sustained elevation of SMAD1/5 phosphorylation (Figure 2E,F). All concentrations tested (IL‐6: 1 and 10 ng/mL; hepcidin: 0.1 and 1 µg/mL) showed significant increases in SMAD1/5 phosphorylation compared to control (*p* < 0.01 for IL‐6 10 ng/mL, *p* < 0.01 for hepcidin 1 µg/mL).

This sustained SMAD1/5 activation was accompanied by increased transcription of inhibitors of differentiation (ID) proteins. IL‐6 treatment significantly increased mRNA levels of ID1 (1.7‐fold, *p* < 0.05), ID2 (2.2‐fold, *p* < 0.05), ID3 (2.1‐fold, *p* < 0.05), and ID4 (1.8‐fold, *p* < 0.05) (Figure 2G–K). Hepcidin treatment showed more selective effects, significantly increasing ID1 expression (2‐fold, *p* < 0.05) but having less pronounced effects on other ID family members. These transcriptional changes were reflected at the protein level, with both IL‐6 and hepcidin treatments leading to significant increases in ID1 protein expression after 24 h (IL‐6: 1.5‐fold and 1.6‐fold for 1 and 10 ng/mL respectively, *p* < 0.001; hepcidin: 2.2‐fold and 2.1‐fold for 0.1 and 1 µg/mL respectively, *p* < 0.01) (Figure 2L,M).

### 3.3 BMPR2 Knockdown Effects on Downstream Signalling

**Figure 3:**
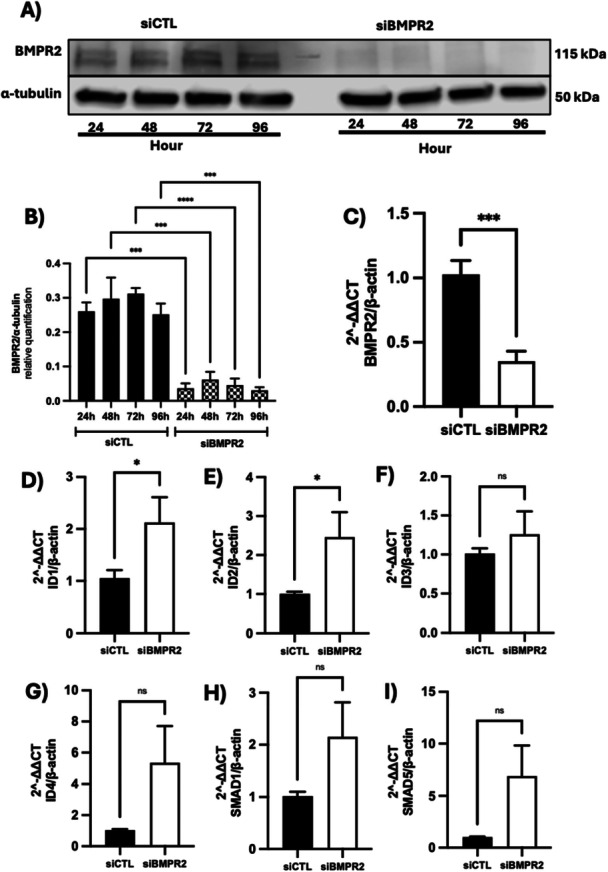
Time‐course of BMPR2 knockdown in hPAECs and its effects on downstream signaling pathways. (A) Western blot analysis of protein lysates from hPAECs over time. Lysates were collected at 24, 48, 72, and 96 h after transfection with either control siRNA (left) or BMPR2 siRNA (right). (B) Relative quantification of BMPR2 protein levels using ImageJ. Data are presented as mean ± SEM (*n* = 3). Statistical significance was determined by one‐way ANOVA followed by Dunnett's post hoc test (***p < 0.001, ****p < 0.0001). (C) BMPR2 mRNA levels 24 h post‐knockdown. Data are presented as mean ± SEM (*n* = 6). Statistical significance was determined by an unpaired *t*‐test (***p < 0.001). (D–I) mRNA levels of downstream targets ID1 (D), ID2 (E), ID3 (F), ID4 (G), SMAD1 (H), and SMAD5 (I) 24 h post‐knockdown. Data are presented as mean ± SEM (*n* = 6). Statistical significance was determined by unpaired *t*‐tests (*p < 0.05).

To determine whether the observed signalling changes were directly due to BMPR2 loss, we performed siRNA‐mediated BMPR2 knockdown in hPAECs. Western blot analysis demonstrated effective knockdown of BMPR2 protein expression over a 96‐h time course (Figure 3A). Densitometric quantification showed significant reduction in BMPR2 protein levels at all time points examined (24, 48, 72, and 96 h post‐transfection) compared to siRNA control (*p* < 0.001 at 24 h, *p* < 0.0001 at 48 h, *p* < 0.001 at 72 h, *p* < 0.001 at 96 h) (Figure 3B). At the mRNA level, BMPR2 expression was reduced by approximately 70% at 24 h post‐transfection (*p* < 0.001) (Figure 3C).

Analysis of downstream targets revealed that BMPR2 knockdown significantly increased ID1 and ID2 mRNA levels (2.1‐fold increase, *p* < 0.05 and 2.4‐fold increase, *p* < 0.05, respectively) (Figure 3D,E). However, effects on ID3 and ID4 expression were not statistically significant (Figure 3F,G). Similarly, while there was a trend toward increased SMAD1 and SMAD5 mRNA levels following BMPR2 knockdown, these changes did not reach statistical significance (Figure 3H,I).

These findings demonstrate that BMPR2 knockdown partially recapitulates the effects of IL‐6 and hepcidin treatment, particularly regarding ID1 and ID2 regulation, suggesting both BMPR2‐dependent and independent mechanisms may be involved in the response to these factors.

## 4 Discussion

This study demonstrates that IL‐6 and hepcidin, both implicated in iron homeostasis and inflammation, significantly reduce BMPR2 expression in hPAECs at both the mRNA and protein levels. This aligns with earlier findings implicating IL‐6 in BMPR2 downregulation via STAT3‐dependent mechanisms [16]. IL‐6 suppresses BMPR2 expression, also true for hepcidin, likely through feedback involving BMP‐SMAD signalling.

Intriguingly, despite reduced BMPR2 levels, downstream signalling via SMAD1/5 remains active, as evidenced by increased phosphorylation. These findings are consistent with the hypothesis that alternative receptor complexes can compensate for BMPR2 deficiency, maintaining signalling through heteromeric BMP/TGF‐β [7]. This compensatory mechanism may explain the paradoxical increases in ID protein expression observed after BMPR2 reduction. The therapeutic implications of this finding are particularly relevant given the recent success of sotatercept in PAH treatment [17]. Sotatercept acts as a ligand trap for TGF‐β superfamily members, including activin A and growth differentiation factors [18], potentially rebalancing signalling in the context of reduced BMPR2 [19]. Our findings suggest that targeting these alternative pathways might be particularly effective in contexts where IL‐6 or hepcidin levels are elevated.

**Figure 4:**
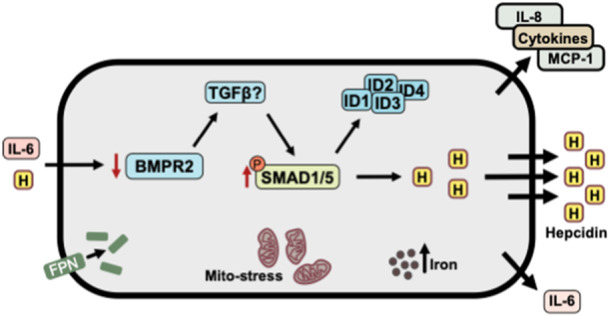
Potential role of hepcidin in hPAECs in the development of PAH. Elevated serum hepcidin levels have been observed in PAH patients. Hepcidin downregulates BMPR2, yet paradoxically increases SMAD1/5 phosphorylation, leading to elevated expression of ID proteins (ID1, ID2, ID3, and ID4) and further hepcidin production. This signalling could be mediated through the activation of other TGF‐β receptors to compensate for the loss of BMPR2. The upregulation of hepcidin promotes ferroportin degradation and internalization, which can result in iron accumulation within the cell, generating mitochondrial stress (mito‐stress). Increased iron retention has been associated with the activation of transcription factors, which stimulate the release of inflammatory markers, such as IL‐6, IL‐8, and MCP‐1. IL‐6, in turn, acts as an agonist of hepcidin, potentially creating a feedback loop that leads to further hepcidin synthesis.

Together with previous work, where we have demonstrated iron accumulation, mitochondrial stress, hepcidin, IL‐6 and cytokine release in response to hepcidin stimulation [9, 10, 11] we present a potential role for hepcidin in hPAECs in the context of PAH (Figure 4). In this pathway, elevated serum hepcidin levels, observed in PAH patients, trigger downregulation of BMPR2 while increasing SMAD1/5 phosphorylation through compensatory activation of alternative TGF‐β receptors. This leads to elevated expression of ID proteins (ID1‐4) and subsequent hepcidin production. Concurrently, hepcidin promotes ferroportin degradation and internalisation, resulting in cellular iron accumulation and mitochondrial stress. This iron dysregulation stimulates the release of inflammatory markers, including IL‐6, IL‐8, and MCP‐1, with IL‐6 acting as a hepcidin agonist to potentially perpetuate this pathogenic cycle. This mechanistic insight connects our current findings of maintained SMAD1/5 signalling despite BMPR2 reduction with our previous observations of iron‐mediated cellular dysfunction in PAH pathogenesis.

### 4.1 Implications for PAH Pathogenesis

The link between BMPR2 mutations and PAH is well established, with BMPR2 loss contributing to endothelial dysfunction, smooth muscle cell proliferation, and vascular remodelling [3]. Our findings suggest that IL‐6 and hepcidin exacerbate these processes by directly downregulating BMPR2, thereby promoting pathological signalling cascades. The upregulation of ID proteins, particularly ID1 and ID3, aligns with prior reports linking these factors to enhanced cell growth and metastasis [20, 21].

We have previously demonstrated variation in hPAEC and hPASMC response to hepcidin treatment, with hPASMCs exhibiting relatively increased proliferation, migration, and mitochondrial dysfunction under hepcidin challenge [9]. This cell‐type‐specific response is important when considering the pathophysiology of PAH. Previous work has shown increased Id1 expression following BMPR2 loss in endothelial cells [22, 23]. Notably, Hiepen et al. (2019) found that in BMPR2‐deficient endothelial cells treated with BMP6 (a key ligand for hepcidin synthesis), Id3 was upregulated [7]. In contrast, BMPR2 loss in hPASMCs reduced Id1 and Id3 protein levels, with Id1 loss associated with increased hPASMC proliferation [24, 25]. Taken together, variable responses in cell type when challenged with mediators of BMP and ID signalling, may begin to elucidate PAH progression mechanics.

Future studies should explore whether ID protein knockdown can prevent the proliferative effects of IL‐6 and hepcidin treatment. This would help determine whether ID proteins are necessary mediators of the pathological effects of these factors or merely markers of pathway activation. The differential effects of BMPR2 loss in endothelial versus smooth muscle cells warrant further investigation, as previous studies have shown reduced ID1 and ID3 levels in BMPR2‐deficient pulmonary arterial smooth muscle cells [26].

### 4.2 Therapeutic Insights

Our results highlight potential therapeutic avenues targeting the IL‐6‐BMPR2‐hepcidin axis in PAH. Strategies aimed at restoring BMPR2 function or rebalancing BMP/TGF‐β signalling could mitigate the pathological effects of IL‐6 and hepcidin. The success of sotatercept in recent clinical trials (PULSAR and STELLAR studies REFS) demonstrates the potential of this approach, with significant improvements in 6‐min walk distance and reduction in pulmonary vascular resistance [17]. Our findings suggest that sotatercept's efficacy might be particularly pronounced in patients with elevated IL‐6 or hepcidin levels, as these factors appear to drive signalling through alternative TGF‐β pathways that sotatercept can effectively target.

## 5 Conclusion

Our findings provide new insights into the regulatory interplay between IL‐6, hepcidin, and BMPR2 in the context of PAH. Both IL‐6 and hepcidin downregulate BMPR2 expression, yet downstream signalling through SMAD1/5 persists, likely due to compensatory activation of alternative receptor complexes. This paradox highlights the complexity of BMP/TGF‐β signalling in vascular remodelling and underscores the multifaceted role of BMPR2 in endothelial cell function. Future studies should focus on identifying the specific alternative receptor complexes involved and determining whether targeted inhibition of ID proteins could prevent pathological vascular remodelling in PAH.

## Author Contributions

Quezia K. Toe performed all experiments, generated and analysed the data, prepared all figures, and drafted the initial manuscript. Theo Issitt contributed to manuscript writing, figure arrangement, laboratory support, and critical revision of the manuscript. Gregory J. Quinlan conceived the study, developed the study design, supervised the research, and critically revised the manuscript for important intellectual content. S. John Wort contributed to study conception, supervised the research, and critically revised the manuscript. All authors contributed to data interpretation, reviewed and approved the final version of the manuscript, and agree to be accountable for all aspects of the work.

## Ethics Statement

The use of normal lung tissue has been approved by the Royal Brompton and Harefield NHS Trust Research Ethics Committee (ethics number GQJW1). All procedures were carried out in accordance with the relevant guidelines and regulations. All patients gave written, informed consent before the use of their lung tissue.

## Conflicts of Interest

The authors declare no conflicts of interest.

## Supporting information

SuppGels.

## Data Availability

The data sets generated and analysed during the current study are available from the corresponding author upon reasonable request.
