# Supplementary material for: The Interplay Between IL‐6, Hepcidin, and BMPR2 Signalling in Pulmonary Arterial Hypertension: Mechanistic Insights Into Vascular Remodelling
Source: Pulm Circ. 2026 Apr 5;16(2):e70272. doi: 10.1002/pul2.70272 (PMC13051983; doi:10.1002/pul2.70272)
Supplement: Supplementary file 1 — SuppGels. [file PUL2-16-e70272-s001.pdf]

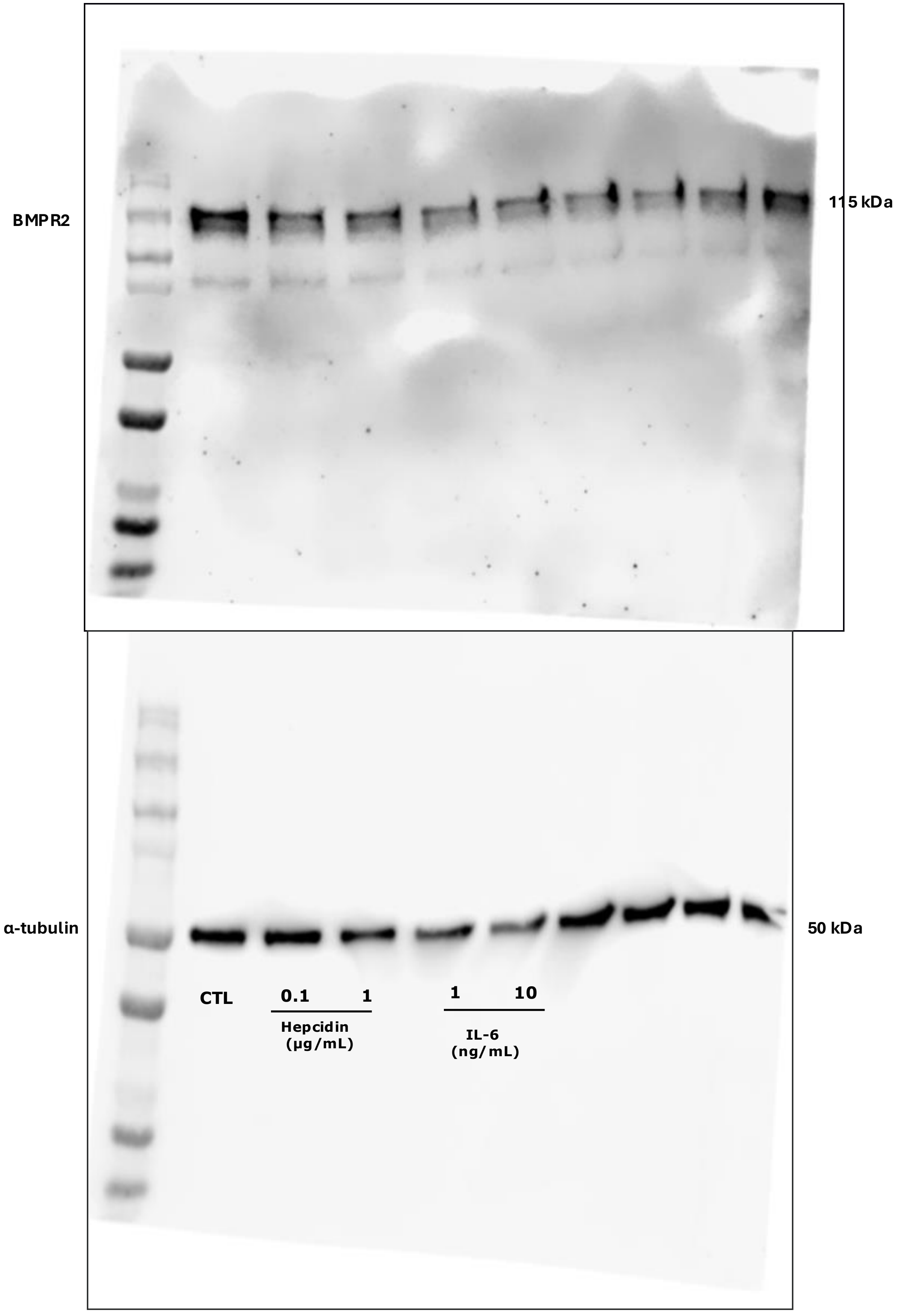

Supplementary figure 1. Uncropped western blot gels from Figure 1.

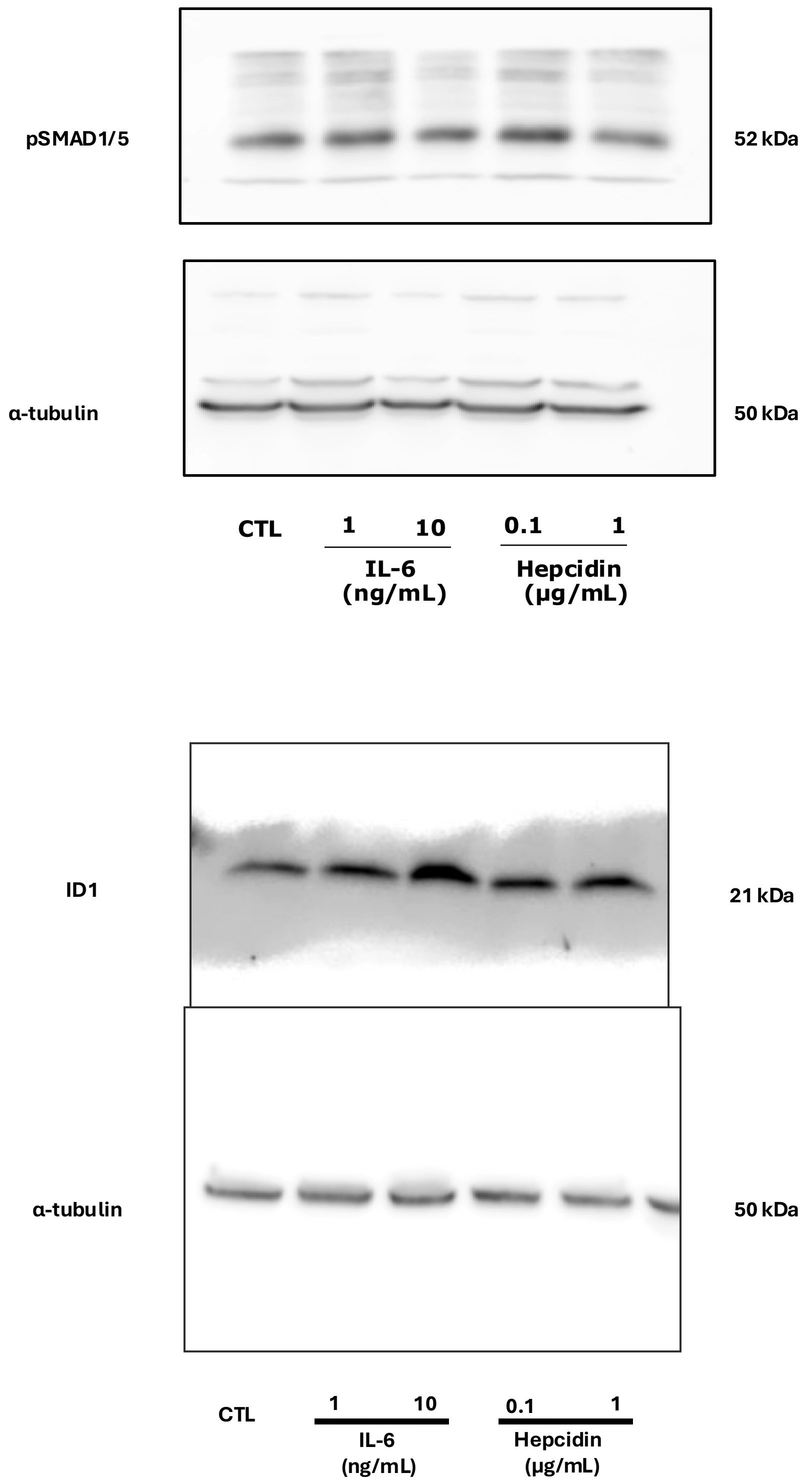

Supplementary figure 1. Uncropped western blot gels from Figure 2.

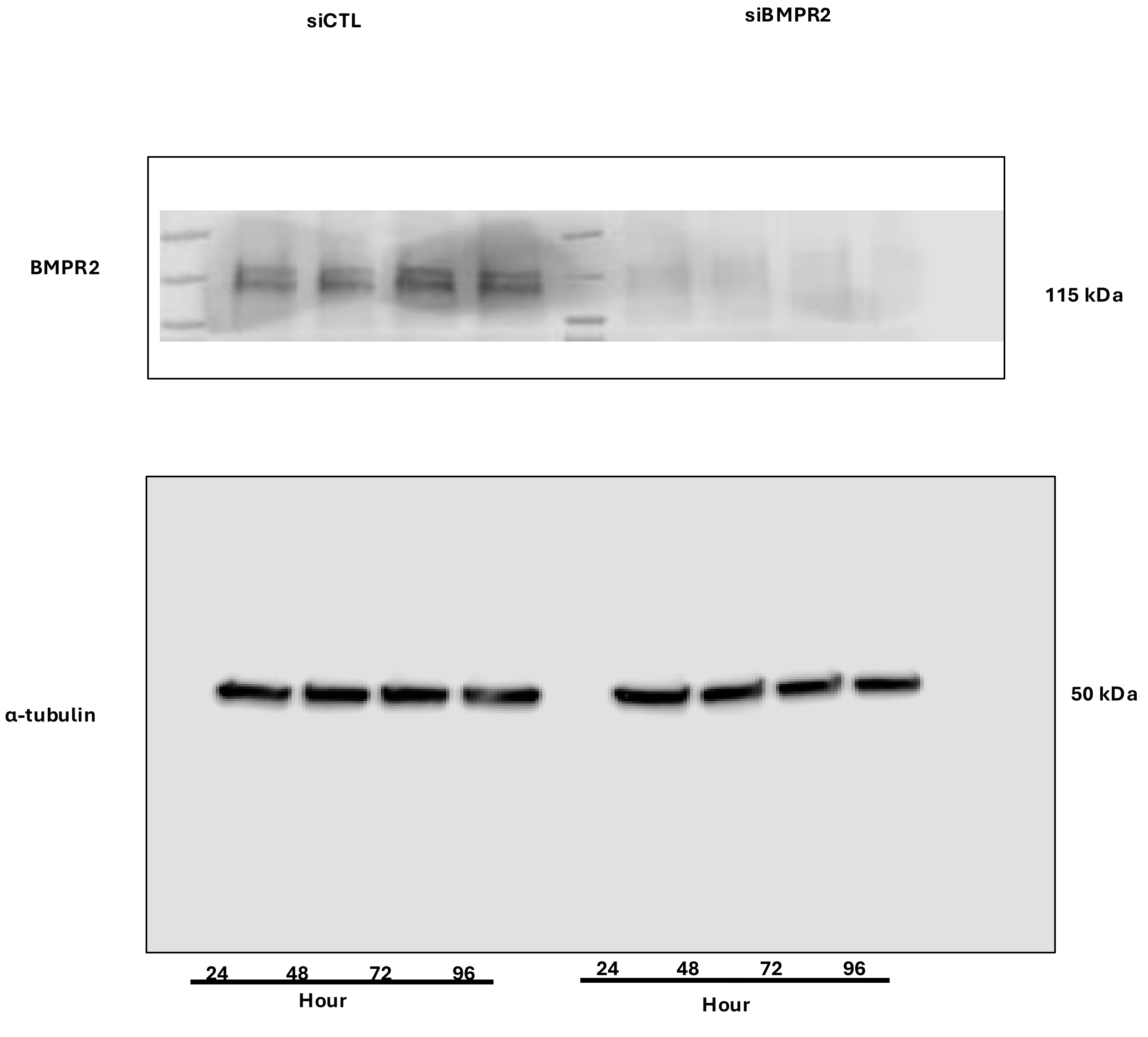

Supplementary figure 1. Uncropped western blot gels from Figure 3.
